# Safety and effectiveness of rivaroxaban thromboprophylaxis in adrenocorticotropic hormone–dependent Cushing syndrome

**DOI:** 10.1210/clinem/dgag014

**Published:** 2026-01-16

**Authors:** Zin Htut, Aastha Mundhra, Katharine Lazarus, Niamh Martin, Debbie Papadopoulou, Karim Meeran, Florian Wernig

**Affiliations:** Division of Diabetes, Endocrinology and Metabolism, Imperial College London, London W12 0NN, UK; Division of Diabetes, Endocrinology and Metabolism, Imperial College London, London W12 0NN, UK; Division of Diabetes, Endocrinology and Metabolism, Imperial College London, London W12 0NN, UK; Division of Diabetes, Endocrinology and Metabolism, Imperial College London, London W12 0NN, UK; Department of Endocrinology, Imperial College Healthcare NHS Trust, London W12 0HS, UK; Department of Endocrinology, Imperial College Healthcare NHS Trust, London W12 0HS, UK; Division of Diabetes, Endocrinology and Metabolism, Imperial College London, London W12 0NN, UK; Department of Endocrinology, Imperial College Healthcare NHS Trust, London W12 0HS, UK; Division of Diabetes, Endocrinology and Metabolism, Imperial College London, London W12 0NN, UK; Department of Endocrinology, Imperial College Healthcare NHS Trust, London W12 0HS, UK

**Keywords:** Cushing syndrome, venous thromboembolism, rivaroxaban, thromboprophylaxis

## Abstract

**Context:**

Adrenocorticotropic hormone (ACTH)–dependent Cushing syndrome (CS) is associated with a markedly increased risk of venous thromboembolism (VTE), yet thromboprophylaxis strategies remain inconsistent across clinical practice. In 2019, our center introduced routine oral rivaroxaban prophylaxis (10 mg once daily) for all patients with ACTH-dependent CS.

**Objective:**

To evaluate the safety and effectiveness of routine rivaroxaban prophylaxis in ACTH-dependent CS.

**Methods:**

We retrospectively reviewed 70 adults with ACTH-dependent CS managed between 2012 and 2025 (29 pre-2019 and 41 post-2019) to compare VTE incidence before and after the introduction of rivaroxaban.

**Results:**

There were no differences in baseline characteristics between the 2 groups. Cushing disease (ie, secondary to a pituitary corticotroph adenoma) was the most common subtype (26/29 pre-2019 and 34/41 post-2019). Among patients without routine prophylaxis (pre-2019), 4 patients (13.8%) developed 6 VTE events, occurring both pre- and postoperatively. In the post-2019 cohort, 5 patients experienced 7 VTE events before endocrine assessment and before rivaroxaban could be initiated. Two had recurrent VTE and were already receiving long-term treatment-dose anticoagulation; therefore, 39 of the 41 proceeded with rivaroxaban prophylaxis. No major or minor bleeding complications were observed. Hematological parameters remained stable, and all patients completed the prescribed prophylaxis course (median duration: 7.9 months).

**Conclusion:**

In our cohort, VTE incidence was 13.8% without prophylaxis and did not occur after the introduction of routine rivaroxaban. Prophylactic oral rivaroxaban in ACTH-dependent CS was safe and effective in preventing new, recurrent, perioperative, and postoperative VTE events, supporting its early initiation at diagnosis and continuation through the peri- and postoperative period.

Cushing syndrome (CS) is a rare endocrine disorder caused by chronic exposure to excess glucocorticoids. Exogenous (iatrogenic) glucocorticoid excess accounts for most cases overall, whereas endogenous CS results from autonomous cortisol hypersecretion—most commonly due to an adrenocorticotropic hormone (ACTH)–secreting pituitary adenoma (Cushing disease [CD]) or ectopic ACTH production from nonpituitary tumors (ectopic CS) (1). In addition to its well-known metabolic, cardiovascular, and neuropsychiatric complications, endogenous CS is associated with a significant increase in the risk of venous thromboembolism (VTE), including deep vein thrombosis (DVT) and pulmonary embolism (PE) (2, 3).

The prothrombotic state in CS is multifactorial (4, 5). Excess cortisol contributes to hypercoagulability through upregulation of procoagulant factors (such as fibrinogen, factor VIII, and von Willebrand factor), suppression of fibrinolysis via increased plasminogen activator inhibitor-1, and endothelial dysfunction. These changes collectively alter the balance of the coagulation cascade in favor of clot formation. A shortened activated partial thromboplastin time, frequently observed in CS, also reflects increased activation of the coagulation cascade (6). In addition, increased platelet activation and reduced anticoagulant proteins (protein C and S) may further contribute to thrombotic risk (7). Notably, this hypercoagulable state may persist even after biochemical remission and is particularly pronounced during active hypercortisolism and the perioperative period (6).

Despite this recognized risk, routine thromboprophylaxis is not universally implemented in clinical practice, and guidelines remain variable (8, 9). It is important to recognize 2 distinct clinical settings in which thromboprophylaxis should be considered in CS: (1) during active hypercortisolism, where reported VTE incidence ranges from 5% to 20% (10-12), and (2) the postoperative period, where VTE rates are typically 2% to 5% despite standard perioperative prophylaxis, rising to over 10% in patients with more severe disease or additional risk factors (13-15). However, these estimates may be conservative due to retrospective study designs and underdiagnosis of asymptomatic events.

A 2025 international Delphi consensus recommended pharmacological thromboprophylaxis for all patients with active CS, initiated at diagnosis and continued until remission (16).

In 2019, our center introduced routine prophylaxis with oral rivaroxaban 10 mg once daily for patients with ACTH-dependent CS (pituitary and ectopic), who typically exhibit more severe and prolonged hypercortisolism. Prospective data also suggest that adrenal CS is associated with a less prothrombotic profile than ACTH-dependent disease, supporting our decision not to implement routine prophylaxis in this subgroup (17). We conducted a retrospective cohort study comparing VTE incidence and safety outcomes before and after implementation of rivaroxaban prophylaxis.

## Material and methods

### Study design and population

This single-center retrospective study included adults (≥18 years) with confirmed ACTH-dependent CS managed at Imperial College Healthcare NHS Trust between January 2012 and January 2025. Patients with exogenous glucocorticoid exposure or incomplete records were excluded.

Cushing disease was diagnosed based on elevated ACTH levels, identification of a pituitary adenoma on magnetic resonance imaging, and confirmation of a central-to-peripheral ACTH gradient on inferior petrosal sinus sampling (IPSS), where performed. Ectopic CS was defined as elevated ACTH without a central-to-peripheral gradient on IPSS and localization of a nonpituitary source of ACTH secretion on imaging.

### Anticoagulation protocol

Before 2019, our center did not have a standardized thromboprophylaxis protocol, and pharmacological prophylaxis was generally not administered to patients with ACTH-dependent CS, including those with ectopic ACTH production.

The decision to introduce a formal protocol in 2019 was prompted by accumulating evidence of high VTE risk in CS (including the studies by Boscaro et al [18] and Barbot et al [19]) together with several VTE events observed in our own cohort, which highlighted the need for a more consistent institutional approach. As a large tertiary referral center managing many patients with severe and prolonged hypercortisolism, we therefore adopted a proactive strategy aimed to reducing thrombotic complications.

In 2019, we implemented a local protocol recommending routine thromboprophylaxis for all patients with ACTH-dependent CS. The standard regimen was rivaroxaban 10 mg orally once daily, initiated at diagnosis and continued until 3 months posttranssphenoidal surgery, unless contraindicated (eg, thrombocytopenia or other bleeding risks). Rivaroxaban was withheld 48 hours before interventional procedures—including IPSS and surgery—and restarted 24 hours after IPSS or 48-72 hours after surgery in the absence of bleeding. Patients with a prior history of VTE were included in the study; those requiring anticoagulation for indications unrelated to CS (eg, atrial fibrillation, prosthetic heart valves) were excluded.

### Data collection

Data were extracted from electronic medical records. Variables collected included demographics, comorbidities, duration of symptoms, subtype of CS, biochemical markers, and treatment modalities. For patients who developed VTE, data were collected on the number of events, type (DVT or PE), timing relative to diagnosis and surgery, recurrence, and clinical outcome. Additional VTE risk factors—including recent surgery, immobility, long-haul travel, obesity, and other prothrombotic conditions—were also documented. Anticoagulation data included timing of rivaroxaban initiation, duration of therapy, and any associated bleeding complications for safety assessment.

### VTE diagnosis and bleeding risks

Venous thromboembolism was confirmed by imaging (compression Doppler ultrasound for DVT and computed tomography pulmonary angiography for PE). Major bleeding was defined as bleeding that was fatal, involved intracranial or gastrointestinal sites, or required blood transfusion or hospital admission. Minor bleeding was defined as any clinically apparent bleeding that did not meet the criteria for major bleeding.

### Statistical analysis

Descriptive statistics were used to summarize clinical and biochemical data. Continuous variables were presented as mean or median, and categorical variables as counts and percentages. Group comparisons were performed using Fisher's exact test for categorical variables, and *t*-tests or Mann–Whitney *U* tests for continuous variables, as appropriate. A *P*-value of <.05 was considered statistically significant. All analyses were performed using R studio.

### Ethical approval

This study was approved by the Imperial College Healthcare NHS Trust Governance Team as a registered audit. The Governance Team confirmed that the study involved only the use of routinely collected, nonidentifiable clinical data. As such, approval from a research ethics committee was not required under the UK Policy Framework for Health and Social Care Research.

## Results

### Baseline characteristics

A total of 70 patients were included (29 pre-2019 and 41 post-2019). There were no significant differences in baseline characteristics between the 2 groups (Table 1). Comorbidities such as hypertension (70% vs 46%, *P* = .09), diabetes mellitus (41% vs 22%, *P* = .11), obesity (28% vs 10%, *P* = .06), and obstructive sleep apnea (10% vs 5%, *P* = .64) were more frequent in the pre-2019 cohort, although none of these differences reached statistical significance. CD was the most common subtype, with fewer ectopic cases in both cohorts. Metyrapone remained the most frequently used cortisol-lowering medical therapy, while ketoconazole use declined and osilodrostat was introduced post-2019.

**Table 1:** Baseline characteristics, biochemical markers, and treatment modalities in patients with ACTH-dependent CS before and after implementation of routine rivaroxaban prophylaxis

	Pre-2019 cohort (*n* = 29)	Post-2019 cohort (*n* = 41)	*P*-value
Demographics			
Mean age, years	45.5 ± 14.1	45.5 ± 16.9	.99
Female sex, *n* (%)	22 (76%)	34 (83%)	
Mean BMI (kg/m^2^)	33.6 ± 7.5	32.1 ± 6.8	.46
Comorbidities, *n* (%)			
Hypertension	20 (70%)	19 (46%)	.09
Diabetes mellitus	12 (41%)	9 (22%)	.11
Obesity	8 (28%)	4 (10%)	.06
Obstructive sleep apnea	3 (10%)	2 (5%)	.64
Cushing subtype, *n* (%)			
Cushing disease (pituitary)	26 (90%)	34 (83%)	
Newly diagnosed	19 (73%)	31 (91%)	
Recurrent	7 (27%)	3 (9%)	
Ectopic CS	3 (10%)	7 (17%)	
Urinary free cortisol (nmol/24 hours)	1020 ± 866	1166 ± 1629	.73
Hb (g/L)	125 ± 13	122 ± 11	.42
Platelet count (×10^9^/L)	284 ± 59	298 ± 80	.39
Treatment modalities			
Pituitary Cushing disease			
TSS	12 (46%)	16 (47%)	
TSS + adjuvant medical therapy	13 (50%)	18 (53%)	
Metyrapone (*n*)	9	12	
Ketoconazole (*n*)	4	1	
Osilodrostat (*n*)	—	5	
Death, *n* (%)	1 (3%)	—	
Ectopic CS			
Surgical resection	2 (67%)	4 (57%)	
Surgery + medical therapy	1 (33%)	2 (29%)	
Medical therapy alone	—	1 (14%)	

Values are mean ± SD or median (interquartile range) for continuous variables and *n* (%) for categorical variables. Between-group comparisons used *t*-test or Mann–Whitney *U* test (continuous) and Fisher's exact test (categorical), as appropriate.

Abbreviations: BMI, body mass index; Hb, hemoglobin; TSS, transsphenoidal surgery; *n*, number of patients.

### Pre-2019 cohort (no routine thromboprophylaxis)

**Figure 1:**
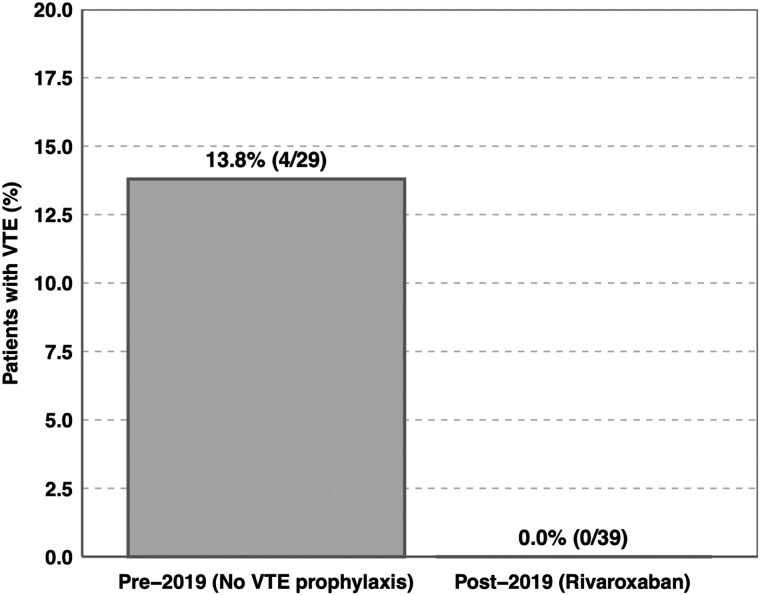
Incidence of VTE in CS before and after rivaroxaban prophylaxis. Bar chart comparing VTE rates in the pre-2019 cohort (no prophylaxis) vs the post-2019 cohort (rivaroxaban 10 mg once daily). VTE occurred in 13.8% of patients before prophylaxis, with 0% events observed after its introduction. Bars display proportions with counts annotated.

Four patients (13.8%) developed a total of 6 VTE events (Fig. 1). VTEs occurred both before (up to 8 months) and within 1 month after surgery (Table 2). Patient 3 experienced recurrent DVT due to subtherapeutic anticoagulation after the first event. Patient 4, who had significant comorbidities, developed bilateral DVT 3 months after diagnosis of CD and died before surgical intervention. Among patients treated with adrenal blocking agents, biochemical remission was achieved in 8/13 (62%).

**Table 2:** VTE events in the pre-2019 cohort (no routine VTE prophylaxis)

Patient	Age (years)	Subtypes	Event type(s)	Location	Timing relative to surgery/diagnosis
1	68	CD	DVT	Right popliteal vein extending through the distal femoral vein into the mid femoral vein	1 month after surgery
2	53	CD	DVT	Left popliteal and posterior tibial veins	8 months before surgery
3	35	CD	DVT ×2	(1) Left lower-limb veins extending from the calf into the external iliac vein (2) Right popliteal vein	2 months before surgery (subtherapeutic anticoagulation) 1 month after surgery
4	75	CD	Bilateral DVT	Bilateral superficial femoral veins	3 months after diagnosis of CD

Listed are patients' age, Cushing subtype (CD/CS), event type (DVT and/or PE), and timing relative to presentation/definite treatment and anatomical site.

Abbreviations: CD/CS, Cushing disease/Cushing syndrome; DVT, deep vein thrombosis; PE, pulmonary embolism.

### Post-2019 cohort (routine rivaroxaban prophylaxis)

Five patients had 7 VTE events before diagnosis of CS and initiation of rivaroxaban (Table 3). Two of these patients had recurrent VTE and were already receiving treatment-dose anticoagulation, and therefore were not included in the prophylaxis group, leaving 39 patients who received rivaroxaban prophylaxis. No VTE events occurred among 39 patients who received rivaroxaban prophylaxis. In this cohort, biochemical remission was achieved in 10/18 (56%) patients treated with adrenal steroidogenesis inhibitors.

**Table 3:** VTE events in the post-2019 cohort occurring prior to endocrine assessment and before initiation of rivaroxaban prophylaxis

Patient	Age (years)	Subtypes	Event type(s)	Location	Timing relative to endocrine contact
1	68	CD	PE	Right segmental and subsegmental pulmonary arteries	48 months before referral
2	31	CD	DVT ×2	(1) Left gastrocnemius vein and (2) posterior tibial veins	36 and 20 months before referral
3	48	CD	DVT and PE	(1) Left popliteal vein and (2) right lower-lobe pulmonary arteries	35 and 16 months before referral
4	26	CD	DVT	Left popliteal and superficial femoral veins	40 months before referral
5	30	Ectopic CS	PE	Right subsegmental pulmonary arteries	12 months before referral

Recorded are patients' age, Cushing subtype (CD/CS), event type (DVT/PE), anatomical location, and timing relative to first endocrine contact.

Abbreviations: CD/CS, Cushing disease/Cushing syndrome; DVT, deep-vein thrombosis; PE, pulmonary embolism.

### Severity of hypercortisolism in patients with VTE

Among patients who experienced VTE before the diagnosis of CS, 24-hour urinary free cortisol (UFC) values ranged from 281 to 1260 nmol/24 hours. In those who developed VTE during active CS, UFC values ranged from 774 to 2052 nmol/24 hours. In comparison, UFC values in patients without VTE ranged from 188 to 2900 nmol/24 hours.

### Bleeding and safety outcomes

Among 39 patients started on rivaroxaban prophylaxis after 2019, no major or minor bleeding episodes occurred. Hematological parameters remained stable throughout follow-up: at diagnosis, mean hemoglobin (Hb) was 128 ± 14 g/L and platelet count 295 ± 72 × 10^9^/L; at preoperative assessment, Hb was 130 ± 13 g/L and platelets 310 ± 76 × 10^9^/L; and at 3 months postoperatively, Hb was 133 ± 13 g/L and platelets 302 ± 71 × 10^9^/L. No bleeding or access-site complications occurred in patients undergoing IPSS, and no cases of pituitary apoplexy were recorded.

### Treatment compliance

All patients completed the prescribed course of rivaroxaban. The median duration of treatment was 7.9 months (range: 4-28 months). No treatment interruptions occurred due to bleeding complications, intolerance, or patient preference.

## Discussion

The implementation of a standardized rivaroxaban prophylaxis was associated with a complete absence of new or recurrent VTE events compared with a 13.8% incidence before its introduction (Fig. 1). Looking at the total cohort from 2012 to 2025 (*n* = 70), 13 events occurred in 9 untreated patients, suggesting that prophylactic anticoagulation is effective in reducing thrombotic complications in this high-risk population. It is important to emphasize 2 distinct clinical settings in which thromboprophylaxis should be considered in CS: during active hypercortisolism and in the postoperative period, both of which are characterized by heightened thrombotic risk.

Comorbidities such as hypertension, diabetes mellitus, and obesity were more prevalent in the pre-2019 cohort and may have contributed to a higher baseline thrombotic risk. Nevertheless, the absence of VTE in the post-2019 cohort, despite the presence of similar clinical risk factors in several patients, suggests that rivaroxaban prophylaxis contributed substantially to reducing VTE events.

Concerns regarding the use of direct oral anticoagulants (DOACs) in CS—given potential bleeding risks, fixed dosing, and lack of routine monitoring—were not substantiated in our cohort (20). Despite cortisol-induced skin and vessel fragility, poor wound healing, and hypertension (21), rivaroxaban was well tolerated, with no bleeding or apoplexy events observed (22). All patients remained compliant with rivaroxaban, and the simplicity of the once-daily oral regimen likely improved adherence. Rivaroxaban prophylaxis was withheld 48 hours before IPSS. Although the 2025 *European Journal of Endocrinology* (*EJE*) position statement advises continuing LMWH around IPSS (Statement 7), it recommends withholding DOACs 24-72 hours prior to the procedure and resuming 48 hours afterwards (16). Our institutional practice of stopping rivaroxaban 48 hours before IPSS is therefore consistent with these recommendations.

Our findings align with prior studies demonstrating that proactive thromboprophylaxis reduces VTE in CS (Table 4 [8, 12, 18, 19, 23]). Most earlier studies evaluated low molecular weight heparin (LMWH), with heterogenous regimens and durations. Boscaro et al (18) demonstrated that extended anticoagulation with unfractionated heparin (UFH) followed by warfarin significantly reduced postoperative VTE, while Barbot et al (19) reported fewer events with 30 days of LMWH compared with shorter courses, without increased bleeding. Similar reductions were observed by Suarez et al (12) with LMWH, and multicenter registry analyses (van Haalen et al [8] and Isand et al [23]) highlighted substantial variation in prophylaxis strategies and the need for standardized protocols. A 2025 Delphi consensus likewise recommends prophylactic-dose LMWH as the preferred agent (16).

**Table 4:** Published literature evaluating thromboprophylaxis in endogenous CS

Study (year)	Design	Population	Prophylaxis/regimens	Timing/duration	Key outcomes	Notes
Boscaro et al (18)	Retrospective cohort, single center	CS (mixed etiologies), *n* ≈ 300	Unfractionated heparin (UFH) × ∼22 days → warfarin ∼4 months (vs no prophylaxis)	Peri and postop; extended prophylaxis	VTE 6% with prophylaxis vs 20% without; *P* < .001	No excess bleeding; first strong evidence for benefit
Barbot et al (19)	Retrospective comparison after TSS	CD post-TSS	Extended LMWH 30 days + compression + mobilization (vs shorter LMWH ≤14 days)	Postop 14 vs 30 days	VTE 0% extended vs 8.8% short (trend, small *n*)	Suggested 30-day LMWH reduces VTE; no bleeding
Suarez et al (12)	Retrospective, single center	CS, mostly surgical (*n* ≈ 200)	Enoxaparin in subset (vs no LMWH)	Peri/postop	Lower VTE with LMWH than without	Not randomized; supports benefit of LMWH
van Haalen et al (8, Endo-ERN survey)	Multinational survey of reference centers	European Endo-ERN	LMWH most common; mechanical prophylaxis also used	Often from diagnosis, periop; duration 2-6 weeks, some up to 3 months	Consensus prophylaxis warranted but highly variable	Highlights need for standard protocols
Isand et al (23)	ERCUSYN registry analysis with surgical subgroup	>1000 patients with CS across Europe	Not standardized; registry data	Perioperative vs nonsurgical periods	High prevalence of VTE, particularly in relation to surgery	Largest multicenter registry evidence; highlights unmet need for uniform prophylaxis strategies

Summary of study design, population intervention (agent and regimen), timing and duration, primary VTE outcome(s), and key findings.

At our center, rivaroxaban was chosen instead because once-daily fixed dosing and avoidance of injections improve practicality and patient acceptability. ERCUSYN registry data indicate wide variability in anticoagulation strategies, with LMWH most commonly used; notably, nearly one-third (30.4%) of VTE events occurred despite prophylaxis, most frequently during LMWH use (68%) (23). By contrast, in our cohort no new or recurrent VTEs occurred under a standardized rivaroxaban protocol, supporting rivaroxaban as a safe, convenient and potentially more effective alternative to LMWH.

Nevertheless, the 2025 *EJE* position statement (Statement 8) recommends LMWH as the preferred thromboprophylactic agent in Cushing's disease, citing the off-label status of DOACs, limited safety data in this population, and the advantage of LMWH's short half-life should bleeding occur (16). Our adoption of rivaroxaban predated this guidance, and although no bleeding complications occurred in our cohort—who did not have additional major bleeding risk factors such as bleeding disorders—further prospective studies are required to define the role of DOACs relative to LMWH in CS.

The optimal duration of postoperative thromboprophylaxis also remains debated. Our center adopted a 3-month course after transsphenoidal surgery, reflecting the persistently elevated VTE risk throughout the postoperative period. In patients with persistent or recurrent hypercortisolism beyond this initial interval, thromboprophylaxis was continued on a case-by-case basis. For patients requiring repeat surgery, prophylaxis was maintained until the re-operation and extended for a further 3 months postafterwards.

Postoperative VTE risk is also relevant in adrenal CS. Babic et al (24) reported VTE events following adrenalectomy. Although the overall risk appears lower than in ACTH-dependent disease, these findings suggest that postoperative thromboprophylaxis should be considered in patients with adrenal CS.

Given that most VTEs in our series occurred before diagnosis, the optimal time to initiate thromboprophylaxis appears to be as soon as ACTH-dependent CS is confirmed, rather than delaying until surgery. In pituitary and ectopic CS, localization of the ACTH source can be challenging, and surgical cure may be delayed. Given diagnostic delays and the protracted exposure to hypercortisolism, early prophylactic anticoagulation may be life-saving.

Notably, VTE occurred even in patients with relatively mild hypercortisolism, with 24-hour UFC values as low as 281 nmol/24 hours. This suggests that thrombotic events are not confined to those with the highest cortisol levels and that even modest biochemical hypercortisolism may confer clinically relevant risk. These observations support consideration of initiating thromboprophylaxis at the time of diagnosis for all patients with ACTH-dependent CS.

### Strengths and limitations

This study has several strengths. All patients received the same rivaroxaban regimen, objective VTE confirmation, and comprehensive safety monitoring. To our knowledge, this is the first study to specifically evaluate bleeding risk with routine oral rivaroxaban prophylaxis in ACTH-dependent CS. The use of a single protocol across the cohort reduces variability and provides practical real-world evidence in this high-risk population.

Limitations include retrospective design, single-center setting, and the possibility that asymptomatic or subclinical VTE events may not have been diagnosed. While the study provides initial evidence on safety and efficacy of rivaroxaban prophylaxis, prospective multicenter studies are warranted to validate these findings and optimize durations and risk-stratified use of DOACs in CS.

### Conclusions

Venous thromboembolism remains a serious complication of ACTH-dependent CS and often develops before diagnosis in the absence of additional risk factors. Oral rivaroxaban prophylaxis (10 mg once daily) was safe, well tolerated, and associated with complete prevention of new or recurrent VTE events in this cohort. Thromboprophylaxis should be considered from the time of diagnosis and continued for an extended postoperative postoperative period of at least 3 months in patients with ACTH-dependent CS.

## Data Availability

The data generated and analyzed in this paper are available upon reasonable request from the corresponding author.
